# Enzymatic treatment of soy protein isolates: effects on the potential allergenicity, technofunctionality, and sensory properties

**DOI:** 10.1002/fsn3.253

**Published:** 2015-06-29

**Authors:** Pia Meinlschmidt, Daniela Sussmann, Ute Schweiggert‐Weisz, Peter Eisner

**Affiliations:** ^1^ Fraunhofer Institute for Process Engineering and Packaging (IVV)Giggenhauser Strasse 35FreisingGermany

**Keywords:** Bitterness, enzymatic hydrolysis, proteolytic enzymes, SDS‐PAGE, soybean allergens

## Abstract

Soybean allergy is of great concern and continues to challenge both consumer and food industry. The present study investigates the enzyme‐assisted reduction in major soybean allergens in soy protein isolate using different food‐grade proteases, while maintaining or improving the sensory attributes and technofunctional properties. SDS‐PAGE analyses showed that hydrolysis with Alcalase, Pepsin, and Papain was most effective in the degradation of the major soybean allergens with proteolytic activities of 100%, 100%, and 95.9%, respectively. In the course of hydrolysis, the degree of hydrolysis increased, and Alcalase showed the highest degree of hydrolysis (13%) among the proteases tested. DSC analysis confirmed the degradation of major soybean allergens. The sensory experiments conducted by a panel of 10 panelists considered the overall improved sensory properties as well as the bitterness of the individual hydrolysates. In particular, Flavourzyme and Papain were attractive due to a less pronounced bitter taste and improved sensory profile (smell, taste, mouthfeeling). Technofunctional properties showed a good solubility at pH 7.0 and 4.0, emulsifying capacity up to 760 mL g^−1^ (Flavourzyme) as well as improved oil‐binding capacities, while the water‐binding properties were generally decreased. Increased foaming activity for all proteases up to 3582% (Pepsin) was observed, whereas lower foaming stability and density were found. The hydrolysates could potentially be used as hypoallergenic ingredients in a variety of food products due to their improved technofunctional properties and a pleasant taste.

## Introduction

Due to its considerable amounts of high‐quality proteins, soy has found wide usage in processed foods during many years. It is applied in numerous food products such as baked, cereal, and meat‐based products as well as hypoallergenic infant formula and vegetarian foods to provide specific functional properties such as improved texture, moisture, fat retention, emulsifying and protein fortification (Sun 2011).

However, one of the major drawbacks of soy‐containing food products is the allergenic potential of soy. Soybean is listed among the “big 8” most allergenic foods comprising those foods that cause 90% of all immunoglobulin E (IgE)‐mediated food allergenic reactions (FDA 2004). Soy allergies can provoke mild symptoms but can also be the cause of life‐threatening reactions, ranging from severe enterocolitis atopic eczema to immediate IgE‐mediated systematic multisystem reactions (Shriver and Yang 2011). Small regions of allergenic proteins, known as epitopes, are responsible for the allergenic reaction by acting with a corresponding antigen (FDA 2004). Even though 42 reactive proteins allergenic proteins have been identified as related to soybean allergy, just the two storage proteins glycinin and *β*‐conglycinin are considered as major soybean allergens (Holzhauser et al. 2009; Amnuaycheewa and de Mejia 2010).

Numerous investigations in the elimination or hypoallergenization of soy ingredients and products have been conducted in recent years. Various thermal and nonthermal processing steps have been applied to combat soybean allergy, including microwave, ultrafiltration, high pressure processing, pulsed ultraviolet light, pulsed electrical fields, irradiation, high intensity ultrasound, genetic or chemical modifications (Shriver and Yang 2011; Verhoeckx et al. 2015). However, most of these methods could not destroy the responsible allergenic epitopes sufficiently or the methods have not yet been investigated in detail.

A more effective approach to reduce the allergenicity of soy proteins is their enzymatic hydrolysis, which has been successfully proven in different studies (Yamanishi et al. 1996; Wilson et al. 2005). Besides the reduction or elimination of the allergenic potential, the destruction of soy proteins due to enzymatic hydrolysis is also accompanied by a loss or change in their functional properties such as solubility as well as foaming, emulsifying, and gelation properties (De la Barca et al. 2000; Ortiz and Wagner 2002; Jung et al. 2004; Tsumura et al. 2005; Yin et al. 2008). In addition, enzymatic hydrolysis could lead to the formation of bitter‐tasting peptides, which also impedes the utilization of hydrolysates in food (Ishibashi et al. 1988; Saha and Hayashi 2001).

Up to now, a feasible technology to reduce soy allergenicity is not implemented in the food industry. As a consequence, total avoidance of soy‐containing products is mandatory to prevent allergenic reactions. However, this is difficult due to the ubiquitous presence of soy proteins in food products. As enzymatic hydrolysis is one of the most effective approaches, it should be investigated in more detail. Former studies have described the effects of proteases either on the level of allergenicity and organoleptic properties or technofunctionality. Literature data about the simultaneous determination of the reduction in the allergenic potential and the alteration of the functional as well as organoleptic properties are not available. This knowledge is a prerequisite for the development of a high‐quality soy‐based food ingredient.

The present study was conducted to (1) assess the effectiveness of different proteases on the degradation of the major soybean allergens (glycinin, *β*‐conglycinin), (2) investigate the effects on the sensory perception with a specific emphasis on the bitter taste, and (3) determine the denaturation profile (DSC) and technofunctional properties of the resulting hydrolysates. The degree of allergenic protein degradation was evaluated and quantified by SDS‐PAGE and by the analysis of the degree of hydrolysis. The organoleptic characteristics with a specific emphasis on the bitter taste were identified. The technofunctional characteristics (protein solubility, emulsifying, foaming, water‐ and oil‐binding capacity) of the obtained hydrolysates have been investigated and their correlation with the observed degradation of the major soybean allergens was examined.

## Material and Methods

### Raw materials and chemicals

Soybeans (*Glycine max* (L.) merr.) were purchased from Naturkost Ernst Weber (Munich, Germany). Enzymes used in this study including Alcalase^®^ 2.4 L FG (endoprotease from *Bacillus licheniformis*), Flavourzyme^®^ 1000 L (endoprotease and exopeptidase from *Aspergillus oryzae*), Protamex^®^ (endopeptidase from *Bacillus licheniformis* and *Bacillus amyloliquefaciens*), Neutrase^®^ 0.8 L (endoprotease from *Bacillus amyloliquefaciens*), and Pancreatic Trypsin Novo^®^ 6.0 S (endopeptidase from Porcine pancreatic glands) were kindly provided by Novozymes A/S (Bagsvaerd, Denmark). Papain (cysteine‐protease from *papaya latex*) (E.C. 3.4.22.2, Sigma no P4762) and Pepsin (endoprotease from Porcine gastric mucosa) (E.C. 3.4.23.1, Sigma no P6887) were purchased from Sigma‐Aldrich Inc. (St. Louis, MO) and Corolase^®^ 7089 (endopeptidase from *Bacillus subtilis*), Corolase^®^ 2TS (endopeptidase from *Bacillus stearothermophilus*) as well as Protease N‐01 (endoprotease from *Bacillus subtilis*) were kindly provided by AB Enzymes GmbH (Darmstadt, Germany) and ASA Spezialenzyme GmbH (Wolfenbüttel, Germany), respectively.

All chemicals used in this study were of analytical grade and obtained from Th. Geyer GmbH & Co. KG (Renningen, Germany).

### Preparation of soy protein isolates (SPI)

Soybeans were dehulled with an underflow peeler (Streckel & Schrader KG, Hamburg, Germany), classified in an air‐lift system (Alpine Hosakawa AG, Augsburg, Germany) and flaked using a roller mill (Streckel & Schrader KG). Soybean flakes were defatted with *n‐*hexane in a percolator (volume 1.5 m^3^, e&e Verfahrenstechnik GmbH, Warendorf, Germany) and flash desolventized with *n*‐hexane (400–500 mbar) prior to steam desolventation. For the preparation of SPI, soy flakes were mixed with acidic water (pH 4.5; 1:8 w/v flakes to water ratio). The suspension was stirred for 1 h at room temperature and separated with a decanter (4400 U min^−1^) for 60 min at 4°C. For protein extraction, the solid phase was stirred in alkaline water (1:8 w/v), which was adjusted to pH 8.0 with 3 mol L^−1^ NaOH. After 60 min of extraction, the suspension was separated (4400 U min^−1^, 60 min) to obtain a clear protein extract, which was adjusted to pH 4.5 with 3 mol L^−1^ HCl (room temperature) to precipitate the proteins. After separation by centrifugation at 5600* g* for 130 min, the isoelectric precipitated protein was neutralized with 3 mol L^−1^ NaOH, pasteurized (70°C, 10 min) and spray dried.

### Enzymatic hydrolysis of SPI

Enzymatic hydrolysis of SPI was performed with different proteases (Table 1) in thermostatically controlled reaction vessels. Therefore, SPI was dispersed in deionized water (5% w/w) utilizing an Ultraturrax for 1 min at 5000 U min^−1^. The obtained slurry was adjusted to enzyme‐specific temperature and pH value (Table 1). After adding the enzyme (E/S‐ratio, see Table 1), the mixture was stirred, maintaining enzymes' optimum temperature and pH value. Aliquots of 100 mL were taken at time intervals of 10, 30, 60, and 120 min to obtain SPI hydrolysates with different degrees of hydrolysis. Reaction conditions for Papain were chosen according to the method of Tsumura et al. (2004). Enzymes were inactivated at 90°C for 20 min in a water bath. Control SPI dispersions were prepared under the same incubation conditions and inactivation treatment, but without enzyme addition. The samples were frozen at −50°C and lyophilized. All experiments were performed in duplicate.

**Table 1:** Degree of hydrolysis (%) of SPI hydrolysates obtained by different protease treatments

Protease	E/S(%)	Temperature(°C)	pH value(−)	Degree of hydrolysis (%)				
				Time of hydrolysis (min)				
				0	10	30	60	120
Alcalase^®^ 2,4L FG	0.5	50	8.0	2.1 ± 0.0^a^	7.5 ± 0.6^b^	9.0 ± 0.5^c^	10.1 ± 0.8^c^	13.0 ± 0.8^d^
Corolase^®^ 7089	0.5	55	7.0	2.1 ± 0.0^a^	5.2 ± 0.3^b^	5.9 ± 0.3^b,c^	5.9 ± 0.5^b,c^	6.8 ± 0.4^c^
Corolase^®^ 2TS	0.5	70	7.0	2.1 ± 0.0^a^	6.8 ± 0.3^b^	7.1 ± 0.2^b^	7.2 ± 0.2^b^	7.8 ± 0.9^b^
Flavourzyme^®^ 1000 L	0.5	50	6.0	2.1 ± 0.0^a^	5.0 ± 0.7^b^	6.1 ± 0.3^b,c^	6.8 ± 0.9^b,c^	8.5 ± 1.1^c^
Neutrase^®^ 0.8 L	0.5	50	6.5	2.1 ± 0.0^a^	3.7 ± 0.1^b^	4.9 ± 1.0^b,c^	5.8 ± 0.7^c^	6.3 ± 0.8^c^
PTN^®^ 6.0 S	0.5	50	9.0	2.1 ± 0.0^a^	2.7 ± 0.1^b^	2.7 ± 0.2^b^	2.7 ± 0.2^b^	2.8 ± 0.1^b^
Papain	0.2	80	7.0	2.1 ± 0.0^a^	4.9 ± 0.0^b^	4.8 ± 0.1^b^	4.7 ± 0.1^b^	4.6 ± 0.4^b^
Papain	0.05	80	7.0	2.1 ± 0.0^a^	3.5 ± 0.2^b^	3.6 ± 0.1^b^	3.8 ± 0.1^b^	3.8 ± 0.3^b^
Pepsin	0.5	50	2.0	2.1 ± 0.0^a^	7.6 ± 0.7^b,c^	7.9 ± 0.4^b^	9.3 ± 0.3^c^	10.6 ± 0.1^d^
Protamex^®^	0.5	60	8.0	2.1 ± 0.0^a^	3.3 ± 0.3^b^	3.9 ± 0.3^b,c^	4.7 ± 0.8^b,c^	5.4 ± 0.8^c^
Protease N‐01	0.5	55	7.2	2.1 ± 0.0^a^	4.3 ± 0.3^b,c^	4.4 ± 0.1^b^	4.5 ± 0.2^b,c^	4.8 ± 0.1^c^

Results are expressed as means ± standard deviation (*n* = 2). Means with different letters within one row indicate significant differences (*P* < 0.05) relating to one protease. Each protease was statistical analyzed separately due to different hydrolysis conditions following ANOVA (Bonferroni).

### Determination of protein degradation due to enzymatic hydrolysis

#### Degree of hydrolysis using the o‐phthaldialdehyde (OPA) method

The degree of hydrolysis (DH) was calculated by determining the free *α*‐amino groups with *o*‐phthaldialdehyde (OPA) using serine as standard (Nielsen et al. 2001).

The percentage of DH was calculated as follows: DH = *h*/*h*_tot_ * 100%; where *h*_tot_ is the total number of peptide bonds per protein equivalent, and *h* is the number of hydrolyzed bonds. The *h*_tot_ factor was 7.8 (based on soy) according to Adler‐Nissen (1986). Six measurements were performed for each sample.

#### Molecular weight distribution applying sodium dodecyl sulfate‐polyacrylamide gel electrophoresis (SDS‐PAGE)

The molecular weight distribution of all samples was determined according to Laemmli (1970) using SDS‐PAGE under reducing conditions. The samples were suspended with 1× Tris‐HCl treatment buffer (0.125 mol L^−1^ Tris‐HCl, 4% SDS, 20% v/v Glycerol, 0.2 mol L^−1^ DTT, 0.02% bromophenol blue, pH 6.8), boiled for 3 min to cleave noncovalent bonds and centrifuged at 12,100 *g* for 4 min (Mini Spin, Eppendorf AG, Hamburg, Germany). The electrophoresis was performed on 4–20% midi Criterion™ TGX Stain‐Free™ precast gels and the proteins were separated using the Midi Criterion™ Cell from Bio‐Rad (Ismaning, Germany). A molecular weight marker (10–250 kDa, Precision Plus Protein™ Unstained Standard, Bio‐Rad Laboratories Inc., Hercules, CA, USA) was additionally loaded onto the gel. Electrophoresis conditions were 200 V, 60 mA, 100 W at room temperature and protein visualization was performed by Criterion Stain‐Free Gel Doc™ EZ Imager (Bio‐Rad).

#### Denaturation profile of the hydrolysates using differential scanning calorimetry (DSC)

The denaturation profiles of all samples were investigated using the differential scanning calorimetry (DSC) method according to Ahmed et al. (2006) with slight modifications using the DSC Q 2000 system from TA Instruments (New Castle, DE). Briefly, the samples were diluted in distilled water to obtain a protein content of 20% (w/w). About 15 mg of the dispersions was weighed into DSC pan. An empty DSC pan was taken as reference. Samples were heated with a heating rate of 2 K min^−1^ in two cycles from 40 to 105°C. All samples were immediately rescanned, after cooling down to 40°C, to investigate reversibility. Peak denaturation temperatures (*T*_*d*_), onset temperatures (*T*_onset_), and relating enthalpies (∆*H*) were calculated by the TA Universal Analysis software. Triplicate determinations were done throughout.

### Chemical composition and technofunctional properties of soy hydrolysates

#### Chemical composition

The chemical composition (protein, ash, and dry matter) was determined as described by AOAC methods (AOAC 2005a,b). The protein contents were calculated based on the nitrogen content (N × 6.25) according to the Dumas combustion method (AOAC 2005b). Dry matter and ash content were analyzed in a thermogravimetrical system (TGA 601, Leco Corporation, St. Joseph, MI) at 105 and 950°C, respectively.

#### Technofunctional properties

##### Emulsifying capacity

The emulsifying capacity (EC) was determined in duplicate as suggested by Wang and Johnson (2001). Protein solution samples of 1% (w/w) were prepared utilizing an Ultraturrax^®^ (IKA‐Werke GmbH & Co. KG, Staufen, Germany) at 18°C. Rapeseed oil was added by a titration system (Titrino 702 SM, Metrohm GmbH & Co. KG, Hertisau, Switzerland) at a constant rate of 10 mL min^−1^ until phase inversion of the emulsion was observed, accomplished by continuous determination of the emulsion's conductivity (conductivity meter LF 521 with electrode KLE 1/T, Wissenschaftlich‐technische Werkstätten GmbH, Weilheim, Germany). The volume of oil needed for phase inversion was used to calculate the EC (mL oil per g sample).

##### Foaming activity, density, and stability

Foaming activity was determined according to Phillips et al. (1987). Protein solution samples (5% w/w) were whipped using the Hobart 50‐N whipping machine (Hobart GmbH, Offenburg, Germany) for 8 min. The relation of the foam volume before and after whipping was utilized for the calculation of the foaming activity. The foaming density was measured by weighing a specified quantity of foam volume. The ratio of foam volume to foam weight was defined as foaming density in g L^−1^. The foaming stability was estimated as the percent loss of foam volume after 60 min.

##### Water‐ and oil‐binding capacity

Water‐binding capacity (WBC) was analyzed according to the AACC 56‐20 official method (AACC 2000). Oil‐binding capacity (OBC) was determined using the method described by Ludwig et al. (1989).

##### Protein solubility

Protein solubility was analyzed at pH 4.0 and 7.0 following the method of Morr et al. (1985). For each pH, 1 g of the sample was suspended in 50 mL 0.1 mol L^−1^ sodium chloride solution. The pH was adjusted using 0.1 mol L^−1^ NaOH or 0.1 mol L^−1^ HCl, while the suspension was stirred at ambient temperature for 1 h. Nondissolved fractions of the samples were separated by centrifugation at 20,000 *g* for 15 min. Afterward, the protein content of the supernatant was determined according to AOAC (2005b).

### Sensory analysis of the protein hydrolysates

#### Training of the panelists

A sensory panel consisted of 10 panelists had been trained for bitterness evaluation over 2 month (1 h per session, twice a week) using the DIN 10959 threshold tests with caffeine solutions at concentrations of 0, 0.025, 0.05, 0.075, 0.1, 0.125, 0.15, 0.175, 0.2, and 0.225 g L^−1^, respectively. Since the bitter profile of caffeine, which was included to select bitter‐taster, is slightly different from a protein hydrolysate solution, an Alcalase hydrolysate was additionally added to the training session. The Alcalase hydrolysate was prepared by incubation of 5% SPI dispersion with 0.5% Alcalase at pH 8.0, 60°C for 3 h without pH adjustment. The hydrolysate was then diluted to obtain solutions of 0.05, 0.1, 0.25, 0.5, 1.0, 1.5, and 2.5 g L^−1^, respectively.

##### Bitter taste evaluation

A 10‐cm line scale anchored from 0 (not detectable) to 10 (intense) was used. For scale calibration, Alcalase hydrolysates with a concentration of 1.0 and 2.5 g L^−1^ were evaluated by the panel to correspond to a bitter intensity of 5 and 10, respectively.

##### Profile analysis

In addition to the determination of the bitter intensity, a profile analysis of the samples was obtained. A broad list of attributes characteristic for the individual samples was developed within the panel. The attributes in terms of smell (“fresh”, “fruity”, “beany”), taste (“sour”, “salty”, “bitter”, “fresh”, “beany”), and mouthfeeling (“mouthcoating”, “astringent”) were also rated on the 10‐cm line scale. The attributes “fresh” and “fruity” are associated with the smell and taste of a lemon, whereas “beany” describes the soybean‐like aroma. “Sour”, “salty”, and “bitter” are associated with fundamental taste sensations elicited by acids, salt, and caffeine, respectively. “Mouthcoating” describes the degree of coating inside the mouth after swallowing, while “astringent” is the trigeminal sensation elicited by grapefruit juice.

#### Sample preparation

Samples were mixed and stirred with tap water to prepare 2.5% (w/w) solutions. This sample concentration was found to be most appropriate for identifying and evaluating the attributes precisely. The pH was adjusted to pH 7.0 with 1 mol L^−1^ NaOH. Each panelist was presented with eight samples (10 mL) per session, which were served to the panel in a random order at room temperature in plastic cups, which were coded by arbitrary numbers (three digits).

#### Sample evaluation

Each sensory evaluation was conducted by the trained panel (performed in 10 sessions, 1 h each). Water and plain crackers were provided for palate cleansing in between. Sensory analyses were carried out in a sensory panel room at 21 ± 1°C. Solutions containing 2.5% SPI, 1.0% and 2.5% Alcalase hydrolysate were prepared as standard for each session, respectively. The assessors were instructed to evaluate bitterness and the attributes mentioned above in relation to the bitterness and attributes of the standard solutions using the standard 10‐cm line scale. Each panelist did a monadic evaluation of the samples at individual speed. Two replicated measurements were made for each sample and replicates were randomized within the same session in order to avoid replicate effects.

### Statistical analysis

All data are expressed as means ± standard deviation of at least two independent measurements (*n* = 2). All chemical data were statistically analyzed by one‐way Analysis of variances (ANOVA) and means were generated and adjusted with Bonferroni post hoc test using SPSS 20.0 (SPSS for windows, SPSS Inc. Chicago, IL). Sensory data (*n* = 10) were also subjected to ANOVA with the use of the Tukey`s HSD average post hoc test. Statistically significant differences were considered at *P *< 0.05.

## Results and Discussion

The enzymatic hydrolysis of SPI, containing a dry matter of 94.4%, a protein content of 94.6%, and an ash content of 4.6%, was conducted in two parts. First, a screening of 10 proteases was carried out. The DH, molecular weight distribution (SDS‐PAGE), and the bitter taste were analyzed to estimate the degradation of the molecules as an indication for the reduction in the allergenic potential. Based on these results, selected proteases were investigated in more detail by determining the denaturation profile (DSC) as well as the technofunctional and sensory (profile analysis) properties.

### Screening of different enzyme preparations

#### Effect of the enzymatic treatment on the protein degradation

##### Degree of Hydrolysis (DH)

The DH gives an initial indication for the change in the molecular integrity and thus for the reduction in allergenic compounds as presented in several studies (Kong et al. 2008; Tavano 2013). During protein hydrolysis, the large complex structured protein molecules are broken down into smaller sized peptides and specific amino acids.

The DH was continuously monitored during enzymatic treatment of SPI. As shown in Table 1, the unhydrolyzed SPI showed an average DH value of 2.1%. In the course of enzymatic hydrolysis, the DH increased significantly (*P* < 0.05). The highest DH value of 13% was achieved after treatment of SPI for 2 h with Alcalase followed by DH values of 10.6%, 8.5%, 7.8%, and 6.8% by using Pepsin, Flavourzyme, Corolase 2TS, and Corolase 7089, respectively. The lowest DH of 2.8% after a 2 h hydrolysis was achieved by Pancreatic Trypsin. This is probably attributed to the presence of the Kunitz Trypsin Inhibitor, inhibiting the proteolytic action of trypsin. The hydrolysis of the proteins was only caused by the enzyme activities as an increase in the DH values could not be observed in the reference experiments (no enzyme addition).

##### Electrophoretic analysis (SDS‐PAGE)

**Figure 1:**
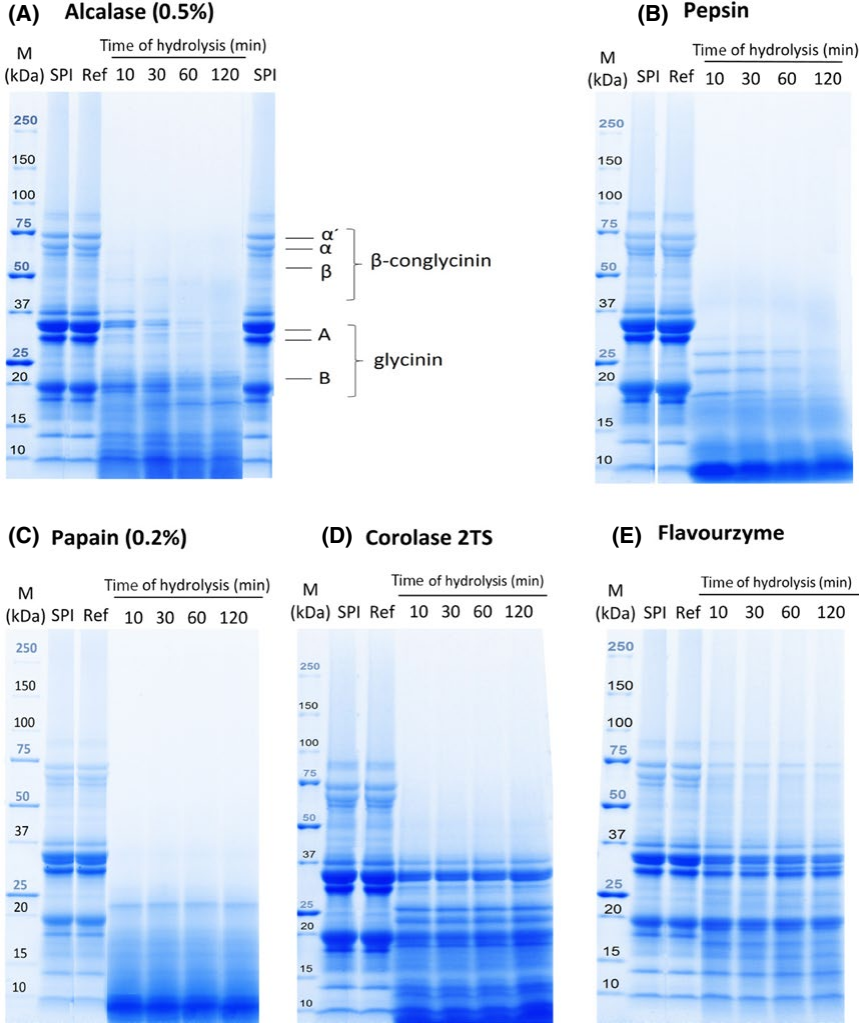
SDS‐PAGE patterns of SPI hydrolysates obtained by different protease treatments*. M* molecular weight standard indicated in kilo Dalton (kDa); *SPI* soy protein isolate; *Ref* reference of each protease (no enzyme addition) after 120 min; Electrophoresis was carried out with 4–20% polyacrylamide gradient gels. For each protein, 50 *μ*g were loaded per well and visualized by UV activation. *α*'‐, *α*‐, and *β*‐ subunits of *β*‐conglycinin; A and B: acidic and basic subunit of glycinin.

A further initial indication for a reduced allergenicity of the hydrolysates was achieved by SDS‐PAGE analyses (Fig. 1A–E). Specific emphasis has been given to the two major soybean allergens (glycinin, *β*‐conglycinin) (Holzhauser et al. 2009; Amnuaycheewa and de Mejia 2010).

In Figure 1, selected SDS‐PAGE profiles are shown exemplarily. The unhydrolyzed SPI and reference (no enzyme addition) presented typical electrophoretic patterns for soy proteins (Fig. 1A). The first three bands are *α*′ (~67–72 kDa), *α* (~63 kDa), and *β* subunits (~47 kDa) of *β*‐conglycinin. Glycinin is composed of two subunits, the acidic subunit “A” (~29–33 kDa) and the basic subunit (“B”) at about 22 kDa (Amnuaycheewa and de Mejia 2010). Already after a 10 min hydrolysis with Alcalase, ß‐conglycinin was completely decomposed, while small amounts glycinin remained still within 30 min of hydrolysis. The acid subunit was eliminated after 60 and 120 min of hydrolysis, respectively, while the basic subunit was not completely destroyed. Similar observations could be obtained by the Pepsin preparation (Fig. 1B). The decreased intensity of the acidic subunit of glycinin was more substantial for proteases such as Alcalase, Pepsin, and Papain than for the other proteases examined. In addition, an increasing reaction time led to a progressive disappearance of the basic subunit. This might be due to the fact that the basic group is located inside the glycinin complex and was therefore less exposed to hydrolysis. In contrast the acidic subunit, which is at the exterior of the complex, was degraded by almost all proteases (Yin et al. 2008).

Pepsin and Papain turned out to be the most effective enzyme preparations (Fig. 1B and C). Already after 10 min of hydrolysis, ß‐conglycinin and glycinin were completely decomposed. A Papain concentration of 0.05% (data not shown) was also examined, which led to similar result as observed for the 0.2% treatment, indicating the high efficiency of Papain (Tsumura et al. 2005). These results were not expected taking the findings of the DH experiments into account as the DH values of the 0.2% and 0.05% Papain hydrolysates were relative low with 4.6% and 3.8% after 2 h, respectively. These differences might be caused by a weak reaction of the OPA reagent with the cysteine residues released during hydrolysis with Papain (cysteine‐protease) (Chen et al. 1979).

The SDS‐PAGE profiles of the hydrolysates obtained by the other enzymes showed a considerable deviating pattern in comparison to Alcalase, Papain, and Pepsin. The SDS‐PAGE profiles of Corolase 2TS and Flavourzyme are shown as an example (Fig. 1D–E) as the SDS‐PAGE profiles obtained by the other enzyme preparations are quite similar (data not shown). It could be shown that these enzymes could slightly deteriorate ß‐conglycinin, but the glycinin subunits remained unchanged. Although Flavourzyme showed only slight changes in the SDS‐PAGE patterns, the DH of 8.5% was contrary high. This might be attributed to the fact that Flavourzyme contains exoproteases, which cleave small peptides at the end of proteins, liberating groups for acting with the OPA reagent.

#### Effects of the enzymatic treatment on the bitterness of SPI

Due to the presence of strongly hydrophobic bitter peptides arising as natural degradation products of proteolytic reactions, enzymatic hydrolysates are often associated with a strong bitter taste (Adler‐Nissen 1986; Ishibashi et al. 1988; Saha and Hayashi 2001; Sun 2011).

Native SPI showed a bitter intensity of 2.8. The bitterness of all hydrolysates increased with increasing reaction time with an exception of the hydrolysate prepared by Flavourzyme, (Table 2). The bitter intensity of the Flavourzyme hydrolysate, increased within the first hour of hydrolysis from initially 2.8 to 4.3, but decreased after 2 h to an intensity of 2.1, which is even lower than the bitterness of native SPI. Flavourzyme contains both endoprotease and exopeptidase activities. The latter can selectively release hydrophobic amino acid residues from the protein molecules, having a debittering effect (Saha and Hayashi 2001).

**Table 2:** Sensory perception (bitterness) of hydrolyzed SPI obtained by different protease treatments

Protease	Intensity of bitterness				
	Time of hydrolysis (min)				
	0	10	30	60	120
Alcalase^®^ 2,4L FG	2.8 ± 0.9^a^	8.7 ± 1.4^b^	8.6 ± 1.5^b^	9.2 ± 0.8^b^	9.2 ± 1.1^b^
Corolase^®^ 7089	2.8 ± 0.9^a^	4.5 ± 1.2^a^	4.7 ± 1.5^a,b^	6.9 ± 1.2^b,c^	7.6 ± 1.7^c^
Corolase^®^ 2TS	2.8 ± 0.9^a^	6.8 ± 1.7^b^	6.3 ± 1.7^b^	7.0 ± 1.5^b^	7.7 ± 1.8^b^
Flavourzyme^®^ 1000 L	2.8 ± 0.9^a^	4.1 ± 1.2^a,b^	4.5 ± 1.4^b^	4.3 ± 1.8^a,b^	2.1 ± 1.7^a,b^
Neutrase^®^ 0.8 L	2.8 ± 0.9^a^	4.4 ± 1.5^a,b^	4.5 ± 1.4^a,c^	7.0 ± 1.2^b,c^	7.1 ± 0.9^c^
PTN^®^ 6.0 S	2.8 ± 0.9^a^	3.5 ± 1.3^a^	4.0 ± 1.5^a^	3.8 ± 1.8^a^	3.9 ± 1.5^a^
Papain (0.2%)	2.8 ± 0.9^a^	4.3 ± 1.4^b^	4.8 ± 1.1^b^	3.1 ± 1.1^a,b^	3.1 ± 1.5^a,b^
Papain (0.05%)	2.8 ± 0.9^a^	3.3 ± 1.5^a^	3.3 ± 1.9^a^	3.0 ± 1.1^a^	3.0 ± 1.2^a^
Pepsin	2.8 ± 0.9^a^	6.4 ± 1.1^a,b^	4.4 ± 1.6^a,b^	5.4 ± 1.6^a,b^	6.4 ± 1.7^b^
Protamex^®^	2.8 ± 0.9^a^	2.7 ± 1.9^a,b^	4.5 ± 1.6^a,b^	4.6 ± 1.8^a,b^	5.5 ± 1.3^b^
Protease N‐01	2.8 ± 0.9^a^	3.3 ± 1.0^a,b^	6.2 ± 1.1^b,c^	4.4 ± 1.3^a,c^	6.1 ± 1.3^c^

Results are expressed as means ± standard deviation (*n* = 2). Means with different letters within one row indicate significant differences (*P* < 0.05) relating to one protease. Each protease was statistical analyzed separately due to different hydrolysis conditions following ANOVA (Bonferroni).

The highest bitter intensity of 9.2 was achieved using Alcalase followed by Corolase 2TS, Corolase 7089 and Neutrase with bitter intensities of 7.7, 7.6, and 7.1, respectively. The high bitter intensity of the hydrolysates produced by Alcalase is probably caused by the tendency of this enzyme to hydrolyze hydrophobic amino acid residues. Thereby, nonpolar amino acid residues at the C‐terminus of the resulting peptides remain and cause a relatively high bitterness (Adler‐Nissen 1986; Ishibashi et al. 1988; Saha and Hayashi 2001; Sun 2011).

The hydrolysis with 0.2% and 0.05% Papain for 120 min results in low bitterness intensities of 3.1 and 3.0, respectively. Hydrolysis applying the other enzyme preparations resulted in samples with bitter intensities in the range of 5.5 and 6.4 (Table 2).

Among the proteases investigated, Alcalase, Pepsin, and Papain turned out to be most efficient in the degradation of proteins into small‐sized peptides as evidenced by the DH (except Papain) and SDS‐PAGE analysis (Table 1 and Fig. 1), while hydrolysis with Flavourzyme and Papain resulted in hydrolysates with the lowest bitter intensities (Table 2).

#### The effect of enzymatic hydrolysis of SPI on its potential allergenicity, technofunctionality and sensory properties

The most promising enzymes Alcalase, Flavourzyme, Pepsin, Papain, Corolase 7089 as well as Corolase 2TS with respect to a less bitter taste and an effective degradation of molecular weight distribution were analyzed in more detail. The enzymatic hydrolysis was repeated under the same reaction conditions as described in the screening experiments, but the incubation time was changed. Enzymatic hydrolysis with Alcalase, Flavourzyme, Pepsin was performed for 120 min, the treatment with Corolase 7089 and Papain was conducted for 30 min and with Corolase 2TS for 10 min. For Papain, a lower enzyme concentration of 0.05% was applied due to the high reactivity of this enzyme preparation.

#### Effect on the protein degradation

##### Degree of Hydrolysis (DH)

In accordance with the screening trials, Alcalase, Pepsin, and Flavourzyme showed the highest DH values of about 13.6%, 10.0%, and 9.4%, respectively. Lower DH values of about 5.8%, 5.8%, and 3.9% were obtained after hydrolysis with Corolase 7089, Corolase 2TS, and Papain, respectively.

##### Electrophoretic analysis (SDS‐PAGE)

The individual bands of glycinin and ß‐conglycinin units were quantified by Image Lab™ Software (Bio‐Rad, Hercules, CA, USA). The relative hydrolyzation in relation to the unhydrolyzed fractions was calculated (Table 3).

**Table 3:** Degradation of the main allergen fractions of SPI obtained by different protease treatments. Mean values in each column having different letters are significantly different (*P *< 0.05)

	Soy protein isolate (% hydrolyzed)^a^					Total average
	*β*‐conglycinin			Glycinin		
	*α*′	*α*	*β*	A	B	
Alcalase^®^ 2,4L FG	100.0 ± 0.0^a^	100.0 ± 0.0^a^	100.0 ± 0.0^a^	100.0 ± 0.0^a^	100.0 ± 0.0^a^	100.0 ± 0.0^a^
Flavourzyme^®^ 1000 L	61.0 ± 1.0^b^	100.0 ± 0.0^a^	100.0 ± 0.0^a^	27.0 ± 1.0^b^	27.0 ± 2.0^b^	63 ± 0.7^b^
Pepsin	100.0 ± 0.0^a^	100.0 ± 0.0^a^	100.0 ± 0.0^a^	100.0 ± 0.0^a^	100.0 ± 0.0^a^	100.0 ± 0.0^a^
Corolase^®^ 7089	70.5 ± 2.5^c^	100.0 ± 0.0^a^	100.0 ± 0.0^a^	45.4 ± 1.6^c^	35.2 ± 1.4^b^	70.2 ± 0.7^c^
Corolase^®^ 2TS	100.0 ± 0.0^a^	100.0 ± 0.0^a^	100.0 ± 0.0^a^	85.3 ± 1.3^d^	64.5 ± 1.5^c^	90.0 ± 0.5^d^
Papain (0.05%)	100.0 ± 0.0^a^	100.0 ± 0.0^a^	100.0 ± 0.0^a^	100.0 ± 0.0^a^	79.5 ± 0.5^d^	95.9 ± 0.2^e^

*α*,* α*', *β*, subunits of *β*‐conglycinin; A, acidic fraction of glycinin; B, basic fraction of glycinin.

a % of hydrolysis of each main fraction of soy protein isolate treated with different proteases with respect to those of SPI without treatment.

Alcalase, Pepsin, and Papain were the most efficient proteases for the overall degradation of the major allergens with a proteolytic activity of about 100%, 100%, and 95.9%, respectively (Table 3). Alcalase, Corolase 2TS, Pepsin, and Papain hydrolyzed the basic subunit of glycinin with varying degree (Fig. 1 and Table 3). In general, glycinin was least degraded due to its molecular structure and location of the basic subunit, which is covered in the interior of the glycinin complex (Yin et al. 2008).

Hydrolysates prepared with Corolase 7089 and Flavourzyme showed smaller changes in the molecular weight distribution. A complete degradation of the *α* and *β*‐subunits was observed (Table 3), while the *α*′‐subunit was reduced by 70.5% and 61.0%, respectively. However, the acid and basic subunits of glycinin were only slightly affected.

##### Differential scanning calorimetry (DSC)

**Figure 2:**
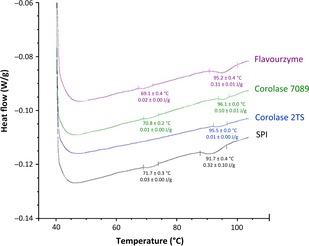
Differential scanning (DSC) thermogram of a 20% unhydrolyzed SPI, Flavourzyme, Corolase 7089, and Corolase 2TS hydrolysates dispersions.

DSC analysis was applied to examine the secondary and tertiary structural changes of SPI due to enzymatic hydrolysis, which can give an additional evidence for the destruction of allergenic proteins. Figure 2 depicts characteristic DSC curves corresponding to unhydrolyzed SPI and three hydrolysates prepared with Flavourzyme, Corolase 7089, and Corolase 2TS while all other hydrolysates exhibit no peaks, indicating complete denaturation of the proteins (data not shown).

SPI showed two endothermic thermal transitions, the major peak denaturation temperatures (*T*_*d*_) were at approximately 71.7°C (*T*_onset_ = 68.8°C) and 91.7°C (*T*_onset_ = 87.0°C) along with denaturation enthalpies of 0.03 and 0.32 J g^−1^, respectively. These results are consistent with previous reports where the onset denaturation temperature of glycinin is around 80–90 and 60–70°C for *β*‐conglycinin (Renkema et al. 2002; Ahmed et al. 2006). Slight variations can be due to genotypic differences in the raw material or varied processing conditions, that is, temperature (Riblett et al. 2001).

The Flavourzyme and Corolase 7089 hydrolysates were likely to be partially denatured or rather partially degraded since the first denaturation point (°C) decreased to 69.1 and 70.8°C, respectively with an enthalpy of 0.02 to 0.01 J g^−1^. The enthalpy of the second denaturation point of the Flavourzyme hydrolysates of about 95.2°C was not significantly (*P *< 0.05) lower compared to native SPI being 0.31 J g^−1^, while a shift of the second denaturation temperature toward higher temperatures was detected. In contrast, the Corolase 7089 hydrolysate exhibited a denaturation point of about 96.1°C with a lower denaturation enthalpy of 0.10 J g^−1^. The Corolase 2TS hydrolysate showed one denaturation temperature at 93.5°C and the enthalpy of denaturation being 0.01 J g^−1^ was significantly (*P* < 0.05) lower than that for SPI, Flavourzyme, and Corolase 7089 hydrolysates. The *β*‐conglycinin fraction was completely denatured, whereas the glycinin complex was only slightly affected as evidenced by a decreased denaturation enthalpy. These findings are in great accordance with the SDS‐PAGE analyses (Table 3).

#### Effects on the sensory profile of SPI

**Figure 3:**
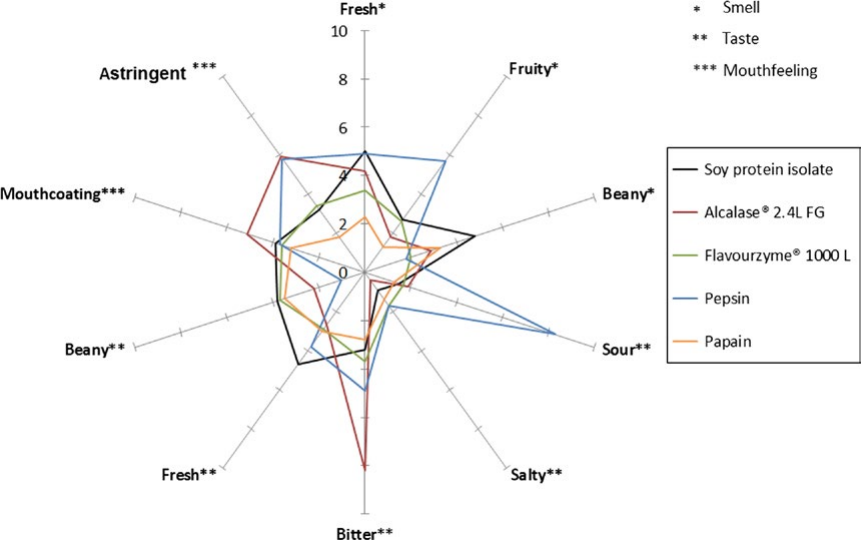
Taste profile (descriptive analysis) of unhydrolyzed SPI, Alcalase, Pepsin, Papain, and Flavourzyme hydrolysates. Each value is expressed as mean ± standard deviation scored on a 10‐cm line scale, ranging from 0 (not detectable) to 10 (intense), by 10 panelists (*n* = 10, 2× replicates), *P* < 0.05 (ANOVA, Tukey's HSD).

Evaluation of the SPI (Fig. 3) by trained panelists resulted in the following smell‐scaling: “fresh” (5.0), “fruity” (2.7), and “beany” (4.8); taste‐scaling: “sour” (1.5), “salty” (0.9), “bitter” (3.2), “fresh” (4.7), and “beany” (3.8); mouthfeeling‐scaling: “mouthcoating” (3.9) and “astringent” (3.2). Compared to SPI, all hydrolysates exhibited a significantly (*P *< 0.05) lower “beany” smell as well as a “fresh” and “beany” taste.

The Alcalase hydrolysate (Fig. 3) showed the highest bitter intensity of 8.2, and therefore, the application of the Alcalase hydrolysate in food systems might be limited. In contrast, the Pepsin hydrolysate (Fig. 3) showed a predominantly “fresh” and “fruity” smell, but the “sour” taste and “astringent” mouthfeeling were significantly (*P* < 0.05) higher than for the other hydrolysates tested. The application of the pepsin hydrolysate as food ingredient might be limited due to its extreme “sour” taste as well as “astringent” mouthfeeling.

The hydrolysate prepared with Papain showed improved sensory properties in comparison to hydrolysates prepared with other proteases tested in terms of “bitter” (2.8) and “beany” taste (3.5), “mouthcoating” (3.2) and “astringency” mouthfeeling (1.8). This sensory profile is even better than the sensory properties of the unhydrolyzed SPI. The sensory properties of the Flavourzyme hydrolysate were comparable to those of native SPI, having the following smell‐scaling: “fresh” (3.4), “fruity” (2.6), and “beany” (2.0); taste: “sour” (1.7), “salty” (1.7), “bitter” (3.7), “fresh” (2.9), and “beany” (3.7); mouthfeeling: “mouthcoating” (3.6) and “astringent” (3.4). The taste profiles of Corolase 2TS and Corolase 7089 were also similar to those of native SPI except of a higher bitter intensity of about 4.9 and 5.3, respectively, and increased “mouthcoating” (4.8 and 5.1) and “astringency” mouthfeeling (4.1 and 3.9) (data not shown).

#### Effects on the technofunctionality of SPI

Technofunctional properties including solubility, gelation, emulsifying, and foaming of proteins connote the physicochemical properties which govern the behavior of protein in the food matrix. Applying enzymatic hydrolysis, functional properties of proteins are modified (Were et al. 1997; De la Barca et al. 2000; Ortiz and Wagner 2002). Enzymatic hydrolysis decreases the molecular weight and increases the number of ionizable groups in proteins and expose hydrophobic groups which change the physical and chemical interactions (Creusot et al. 2006). Soybean proteins glycinin and *β*‐conglycinin mainly reflect the functional properties of SPI and show considerable differences in functional properties such as emulsifying due to their diverse molecular structure (Utsumi and Kinsella 1985).

##### Protein solubility

Solubility is the most important technofunctional property due to its considerable effect on other technofunctional characteristics, particularly gelation, foaming, and emulsifying, which depend on an adequate initial solubility of proteins (Vojdan 1996).

**Figure 4:**
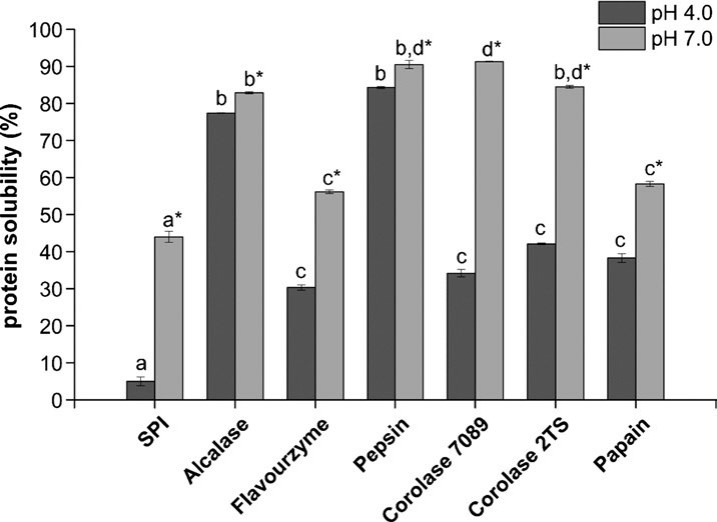
Solubility of SPI and SPI hydrolysates at pH 4.0 and pH 7.0. Means with different letters within one figure indicate significant differences (*P* < 0.05) following ANOVA (Bonferroni). *indicates the solubility at pH 7.0. Results are expressed as means ± standard deviation (*n* = 2).

The solubility of all samples is shown as a function of pH 4.0 and 7.0 in Figure 4. The minimum solubility of 5.0% of the unhydrolyzed SPI was detected at pH 4.0, at the isoelectric point of soybean protein, but was significantly increased after hydrolysis by all proteases. At pH 4.0, the hydrolysates prepared with Alcalase and Pepsin exhibit the highest solubility of 77.4% and 84.3%, respectively. The highest solubility of 91.3% was achieved at pH 7.0 using Corolase 7089 followed by the solubility of 90.5%, 84.5%, and 82.9% by using Pepsin, Corolase 2TS, and Alcalase, respectively. It has been proposed that the reduction in the secondary structure of proteins and the release of smaller peptides, and the corresponding increase in ionizable amino and carboxyl groups are responsible for increased solubility of hydrolysates, increasing the interactions with water molecules (Adler‐Nissen 1986; Ortiz and Wagner 2002). At pH 4.0 the solubility of all other hydrolysates was significantly (*P *< 0.05) lower, ranging from 30.3% to 42.1% and at pH 7.0 between 56.2 and 58.3%.

##### Emulsifying properties

The EC of the unhydrolyzed SPI and hydrolysates was determined. SPI had an EC of 660 mL g^−1^ while all SPI hydrolysates—except hydrolysates generated by Alcalase and Pepsin—showed significantly increased (*P* < 0.05) EC. The Flavourzyme, Corolase 7089, Corolase 2TS, and Papain hydrolysates had EC's of about 760, 730, 670, and 705 mL g^−1^, respectively. Enzymatic hydrolysis has already been used in scientific approaches to improve the emulsifying properties (Wu et al. 1998; Jung et al. 2004). De la Barca et al. (2000) demonstrated an increased emulsification activity after enzymatic hydrolysis of soy protein, which is comparable to the present results. The increased emulsifying properties may be due to the degradation of large protein molecules, exposure of hydrophobic groups and enhanced protein solubility implicating an improved protein surface activity and therefore a better emulsifying activity (Wu et al. 1998). However, the EC of the Alcalase and Pepsin hydrolysates decreased to 438 and 220 mL g ^−1^, respectively, indicating significant (*P* < 0.05) differences compared to unhydrolyzed SPI. The reason for this might be due to the excessive protein hydrolysis; thus, a sharp degradation to smaller peptides as evidenced by DH (Table 1) and SDS‐PAGE results (Table 3). The molecular structure of the protein might be altered, particularly with respect to its interfacial adsorptivity and reduction in continuous phase viscosity, which is essential for the ability to form emulsions (Kinsella et al. 1985).

It has been reported that the EC of hydrolysates is closely related to the degree of hydrolysis, with a low DH (3–5%) increasing and a high DH (~8%) decreasing EC (Achouri et al. 1998). The obtained results in this study cannot entirely confirm these statements. A high DH does not always results in a reduced EC as evidenced by the increased EC of the Flavourzyme hydrolysate, which had a high DH of about 9.4%, but also the highest EC of 760 mL g^−1^.

##### Water‐ and oil‐binding capacity

The WBC of almost all hydrolysates was significantly (*P *< 0.05) lower compared to the unhydrolyzed SPI. The hydrolytic action of proteases causes disruption of the protein network, which is responsible for the inhibition of water‐holding properties. The WBC decreased from an initial WBC of 2.6–1.8 mL g^−1^, 0. 9, and 0.2 mL g^−1^ after hydrolysis with Flavourzyme, Pepsin, and Alcalase, respectively, while no WBC for the Corolase 7089 and Corolase 2TS hydrolysates was observed. However, the Papain hydrolysate showed a significantly (*P *< 0.05) higher WBC with values of 3.9 mL g^−1^.

In contrast, all SPI hydrolysates enhanced the OBC from initially 0.0–3.3 mL g^−1^, 3.3, 3.3, 2.9, 2.8, and 2.1 mL g^−1^ after hydrolysis with Corolase 2TS, Corolase 7089, Papain, Flavourzyme, Pepsin, and Alcalase, respectively. The presence of OBC might be attributed to the exposure of hydrophobic groups after enzymatic hydrolysis allowing the physical entrapment of oil.

##### Foaming properties

The foaming properties are usually characterized in terms of foaming density, activity and stability. Proteins in dispersions cause a lower surface tension at the air–water interface, thus creating a foam (Surowka and Fik 1992). As shown in Table 4, all hydrolysates presented an improved foaming activity. Enzymatic hydrolysis results in smaller peptides with improved foaming activity by rapid diffusion to the air–water interface (Tsumura et al. 2005). Furthermore, native SPI has limited foaming due to its quaternary and tertiary structure, whereas hydrolyzed SPI lost the tertiary structure, which leads to improved foam activity (Yu and Damodaran 1991). Among the proteases studied, the highest foaming activity of 3582% was achieved after hydrolysis with Pepsin, while the hydrolysate prepared with Flavourzyme showed the lowest foaming capacity of 1201% among the hydrolysates. There is an evidence of a trend toward increased foaming activity when the ß‐conglycinin fraction faded and the glycinin fraction becomes dominant, which is supported by SDS‐PAGE profiles (Fig. 1 and Table 3), where only the hydrolysates generated with Flavourzyme and Corolase 7089 showed a slight degradation of the ß‐conglycinin fraction.

**Table 4:** Foaming properties (foaming activity, density, and stability) of SPI and SPI hydrolysates

	Foaming activity (%)	Foaming stability (%)	Foaming density (g L^−1^)
SPI	552 ± 5^a^	90 ± 0^a^	215 ± 5^a^
Alcalase^®^ 2.4L FG	2766 ± 10^b^	0 ± 0^b^	32 ± 0^b^
Flavourzyme^®^ 1000 L	1201 ± 31^a^	86 ± 0^c^	88 ± 3^c^
Pepsin	3582 ± 236^a,b,c^	66 ± 2^d^	27 ± 1^b^
Corolase^®^ 7089	2095 ± 47^b,c^	74 ± 2^a,c,d^	48 ± 1^d^
Corolase^®^ 2TS	2315 ± 111^a,b,c^	68 ± 2^a,c,d^	38 ± 1^b,d^
Papain (0.05%)	2583 ± 0^c^	78 ± 0^a,c,d^	37 ± 0^b,d^

Results are expressed as means ± standard deviation (*n* = 2). Means with different letters within one column indicate significant differences (*P* < 0.05) following ANOVA (Bonferroni).

Although the foaming activities of the hydrolysates were higher compared to SPI, their stability and density decreased (Table 4). The most stable foam was obtained after hydrolysis with Flavourzyme with a stability of 86%, which is near to native SPI with a stability of 90%. For all other hydrolysates, the foaming stability was markedly decreased (Table 4). For foam stabilization some larger protein components are needed, but only few large peptides were found in the hydrolysates, which led to weak foaming stability. The trend of increased foaming activity coupled with decreased foaming stability has been reported in previous studies (Were et al. 1997; De la Barca et al. 2000; Tsumura et al. 2005).

## Conclusion

The aim of this study was to investigate the effect of enzymatic hydrolysis with various proteases on the potential allergenicity, technofunctionality, and sensory properties of SPI. The results clearly demonstrate that enzymatic hydrolysis is an effective approach to reduce the level of allergenicity, while sensory and technofunctional properties can be improved depending on the proteases used. According to the findings, Papain turned out to be the most appropriate proteases for improving the technofunctionality and sensory characteristics, while effectively decreasing the molecular weight of SPI. SDS‐PAGE and the DH were used to examine the degradation the soybean allergens to enable a first evaluation of the level of allergenicity. As this is an indirect method, further research is required to get detailed knowledge of the allergen structure as well as specific and reliable detection methods.

Although the sensory analysis showed promising results, the bitter taste of the produced hydrolysates is still a challenge. Further investigation needs to be carried out focusing on debittering hydrolysates to expand the use in food systems. Studies on enzymatic hydrolysis through various combinations of exo‐ and endopeptidases and other methods for reducing the level of bitterness and allergenicity are ongoing in our laboratory, which might lead to the development of hypoallergenic SPI with pleasant taste and good technofunctionality.

## Conflict of Interest

The authors have declared no conflicts of interest.
